# Principles and Applications of Ultrasonic-Based Nondestructive Methods for Self-Healing in Cementitious Materials

**DOI:** 10.3390/ma10030278

**Published:** 2017-03-10

**Authors:** Eunjong Ahn, Hyunjun Kim, Sung-Han Sim, Sung Woo Shin, Myoungsu Shin

**Affiliations:** 1 School of Urban and Environmental Engineering, Ulsan National Institute of Science and Technology (UNIST), Ulsan 44919, Korea; eunjong@unist.ac.kr (E.A.); guswns3@unist.ac.kr (H.K.); ssim@unist.ac.kr (S.-H.S.); 2 Department of Safety Engineering, Pukyong National University, Pusan 48513, Korea

**Keywords:** self-healing concrete, performance evaluation, nondestructive test, ultrasound

## Abstract

Recently, self-healing technologies have emerged as a promising approach to extend the service life of social infrastructure in the field of concrete construction. However, current evaluations of the self-healing technologies developed for cementitious materials are mostly limited to lab-scale experiments to inspect changes in surface crack width (by optical microscopy) and permeability. Furthermore, there is a universal lack of unified test methods to assess the effectiveness of self-healing technologies. Particularly, with respect to the self-healing of concrete applied in actual construction, nondestructive test methods are required to avoid interrupting the use of the structures under evaluation. This paper presents a review of all existing research on the principles of ultrasonic test methods and case studies pertaining to self-healing concrete. The main objective of the study is to examine the applicability and limitation of various ultrasonic test methods in assessing the self-healing performance. Finally, future directions on the development of reliable assessment methods for self-healing cementitious materials are suggested.

## 1. Introduction

Concrete is one of the most resilient construction materials in the world. However, cracks in concrete due to various reasons may result in serious durability and serviceability problems. Repair and maintenance costs have continuously increased in recent years, and thus, many researchers have tried to develop crack control and self-healing technologies [1,2,3,4,5,6,7,8,9]. Although self-healing concrete construction requires high initial material expenses, it has a very large advantage from the lifecycle cost viewpoint [1].

To satisfy this need for self-healing concrete, researchers have concentrated on the development of engineered self-healing technologies using organic or inorganic chemical agents [10,11,12], microcapsules [13,14,15] and bacteria [16,17,18] over the last decade. Additionally, several studies have examined the use of super absorbent polymer as a potential self-healing agent due to its ability to expand in volume on absorbing water, which can contribute to crack sealing [19,20]. To catalyze the production of crack-filling materials, some researchers used fiber-reinforced concrete or engineered cementitious composites (ECC) and investigated the effect of crack width control on self-healing performance [6,21,22,23,24,25].

With the ongoing development of self-healing technologies for concrete, there is a growing need to develop methods that are able to accurately evaluate the effectiveness of these technologies [26,27,28,29]. Possible evaluation methods corresponding to different self-healing objectives are classified as follows. First, geometric changes (e.g., filling and closing) of surface cracks from self-healing may be visually evaluated by optical microscopy or scanned to a deeper extent by computed tomography (CT) [10,11,14,15,18,20,27,29,30,31,32]. Second, the recovery in the mechanical properties (e.g., stiffness and strength) of self-healing concrete may be evaluated by compression [13,28,33,34], pure tension [21,22,25,35,36,37,38] or bending tests [11,13,17,18,30,39,40,41,42,43,44,45,46]. Third, the change in the durability properties may be assessed by water permeability [12,16,25,26,32], air permeability [47] or ion diffusivity tests [23,24,32]. Finally, the relative change in the material properties due to self-healing may be evaluated through measurements of ultrasound characteristics [11,14,15,18,22,26,27,28,29,30,38,39,40,41,42,43,44,45,46,48], electrical impedance [24] and resonance frequency [23,24]. The development of reliable evaluation methods is important in achieving ultimate success in the development and application of self-healing concrete.

However, current evaluations of the self-healing technologies developed for cementitious materials are mostly limited to lab-scale experiments for inspecting changes in surface crack width (by optical microscopy) and permeability. Furthermore, there are no unified test methods established for assessing the efficiency of self-healing. With respect to the application of self-healing concrete in actual construction, nondestructive test methods are required such that evaluation tasks do not interrupt the use of structures [49,50].

**Figure 1:**
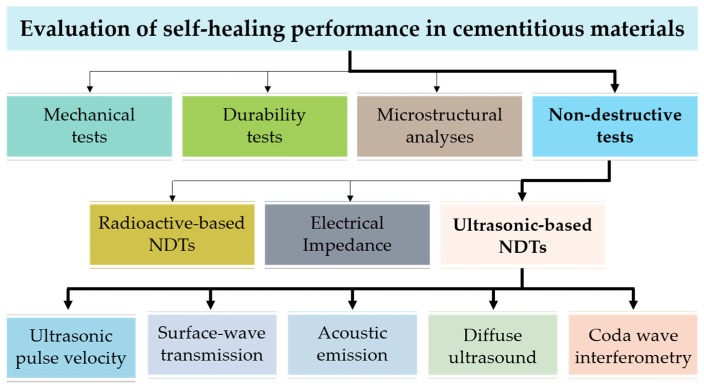
Evaluation of self-healing performance in cementitious materials.

Over the last decade, self-healing performance has been assessed by various ultrasonic nondestructive test methods (Figure 1), which include ultrasonic pulse velocity (UPV) measurement [11,14,15,16,17,18,22,30,33,34,48], surface-wave transmission [26], diffusion in ultrasound [27], coda wave interferometry [28,29] and acoustic emission (AE) [39,40,41,42,43,44,45,46]. However, the applicability and limitation of these test methods for specific cases (e.g., self-healing objectives and types of damage and cracks) are rarely investigated to date.

Given the above-mentioned concerns, the main objective of this study is to examine the applicability and limitation of various ultrasonic test methods in assessing the effectiveness of self-healing technologies developed for cementitious materials. This is performed by thoroughly reviewing the principles of ultrasonic test methods and case studies related to the application of these methods on self-healing concrete. The applicability and limitation of the ultrasonic test methods are analyzed based on the following five criteria: Evaluation of crack size, evaluation of regained mechanical properties (e.g., strength and stiffness), evaluation of regained durability properties (e.g., permeability and chloride-ion diffusivity), appropriate self-healing agents and assessment of in situ structures. Finally, future directions on the research and development of reliable assessment methods for self-healing cementitious materials are proposed.

## 2. Ultrasonic Nondestructive Evaluation Methods

### 2.1. Ultrasonic Pulse Velocity (UPV)

The UPV method is widely used to detect internal defects, and estimate crack depth and compressive strength for concrete structures. Therefore, standard test methods for UPV measurements are well established and are specified in ACI 228.2R [50] and ASTM C597 [51]. According to the standards, the UPV of the first arriving wave (longitudinal wave) is determined through a specific wave path. In this method, transducers with a frequency range between 20 and 100 kHz and a center frequency of 54 kHz are typically used. Two transducers are attached to the surface of concrete, and then the transmission time and velocity of longitudinal waves between the transducers are measured. The transducer arrangement for UPV inspections can be classified into three categories: direct transmission (cross probing), semi-direct transmission, and indirect transmission (surface probing).

**Figure 2:**
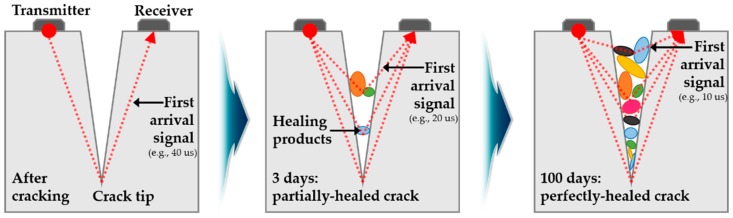
Change in transmission of ultrasonic waves in self-healing process.

Figure 2 shows the change in the travel distance of an ultrasonic wave signal across an initially generated crack and from a partially closed to a fully closed crack. In the evaluation of self-healing using the measurements of UPV, the ratio of relative velocity and change in transmission time may be used as evaluation parameters to estimate the degree of damage, healing ratio and crack depth.

Ferrera et al. [11] evaluated autogenic and engineered self-healing of normal concrete in the presence of crystalline admixtures using relative UPV measurements before damaging and after self-healing. Van Tittelboom et al. [16] investigated the potential of bacteria-based self-healing agents to compare performances of original repair techniques using grout and epoxy. The study [16] compared the change in transmission time before and after self-healing of the cracks. The authors confirmed that there was a recovery of tightness through UPV measurements when using bacteria-based self-healing agents immobilized in silica gel. Williams et al. [18] measured the change in UPV transmission time, with the test results confirming an 8%–30% flexural strength recovery by bacteria-added mortar. Zhu et al. [22] investigated autogenous self-healing of ECC under freeze-thaw cycles damaged by direct tensile tests. Watanabe et al. [48] evaluated self-healing effects in different volumes of fly ash-replaced concrete damaged by freeze and thaw cycles by using ultrasonic tests. These authors utilized the relative amplitude of ultrasonic waves, which is defined as the amplitude of waves normalized after healing with respect to the amplitude of waves in the pure specimen.

Zhong and Yao [33] evaluated the self-healing ability of normal and high-strength concrete damaged under compressive loads at different ages using UPV measurements. Self-healing ratio is defined using compressive strength at the loading and after self-healing. The authors identified that the degree of damage was influenced by the initial strength of the concrete and that the threshold value of normal-strength concrete exceeded that of high-strength concrete. Xu and Yao [17] investigated the performance of non-ureolytic bacteria through pulse velocity. In this study, the previously proposed degree of damage [33] was used, and the healing ratio was defined to quantify the change in UPV. Elmoaty [34] investigated the self-healing efficiency of polymer-modified concretes with different types and doses of polymers and water cement ratios as test parameters using UPV measurements.

Time-of-flight diffraction (TOFD) methods have been used to estimate the recovery of crack depth. This provided evaluations of self-healing performance in cementitious materials with various self-healing mechanisms through microcapsules and impregnation and encapsulation of lightweight aggregates [14,15,30].

### 2.2. Surface-Wave Transmission

**Figure 3:**
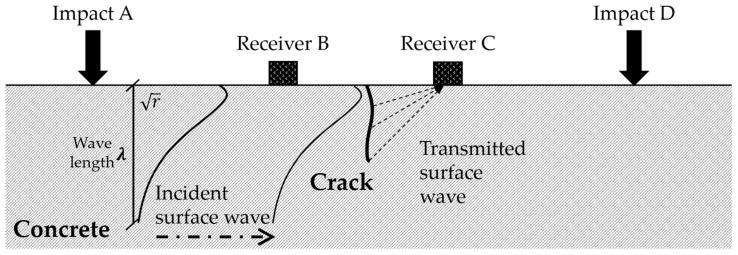
Transmission of surface waves across a crack.

Most of the energy generated by surface impact is transmitted through Rayleigh surface waves instead of body waves [52]. Although the attenuation of body waves, including longitudinal waves (P-waves) and shear waves (S-waves), is proportional to the squared distance to impact sources, the attenuation of surface waves is proportional to the square root of the distance to impact sources. The inspection zone using a Rayleigh wave exceeds that of using body wave sources. A Rayleigh wave is representatively known as a type of surface wave that propagates with elliptic motions combined with horizontal and vertical components. The energy of vertical components of elliptic behaviors is dependent on the height of the propagation waves and thus, the generation of surface waves across a crack is sensitive to crack depth and wavelength. Figure 3 shows the transmission of surface waves across a crack.

Aldea et al. [26] presented the results of self-healing performance evaluation in normal-strength concrete using stress wave transmission and water permeability test. Water permeability tests conducted over 100 days induced continued hydration in the crack surface due to sufficient water supply as well as calcite precipitation from calcium leaching and its reaction with carbonates from water. Real cracks were generated from feedback-controlled splitting tests in 100 by 200 mm cylindrical specimens. Stress waves were generated using solenoid-driven impact sources in a frequency range of 0 to 60 kHz. Transmission of the signal was measured thrice: for uncracked specimens, after crack generation, and after 100 days of water permeability tests. The authors concluded that a large initial crack width resulted in a decrease in signal transmission. Additionally, reduction in permeability coefficients was more significant than the recovery of elastic wave signal transmission that was observed in the process of autogenous self-healing.

Aggelis and Shiotani [53] evaluated the effectiveness of crack repairing by epoxy through both longitudinal and Rayleigh surface wave measurements. The transmission time decreased due to the propagation of the waves across the region filled with epoxy. The change of amplitude and energy of the Rayleigh wave across a partially closed crack could be an important factor for the crack depth. The conventional stress wave techniques support the effectiveness of the Rayleigh wave-based technique in investigating the effects of repair. In addition, Aggelis et al. [54] investigated the efficiency of repair using Rayleigh wave measurements. Single artificial slots with a specific crack depth were prepared at the center of specimen. Then, upper or all parts of the empty slots were filled with epoxy to simulate fully or partially filled slots. Two types of transducers having center frequency with 50 kHz and 115 kHz components were used to describe the effects of wavelength. The amplitude of Rayleigh wave was a good indicator to evaluate the efficiency of repair.

### 2.3. Diffuse Ultrasound

**Figure 4:**
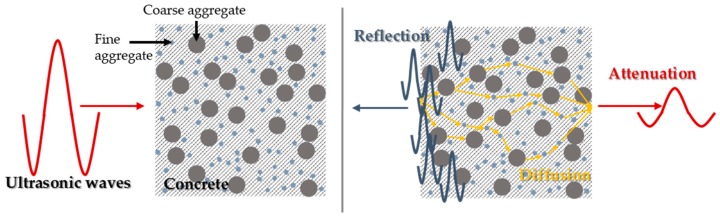
Diffusion of ultrasound in concrete.

The characteristics (e.g., attenuation) of ultrasound propagation are generally dependent on not only the frequency components of propagated waves but also the properties of materials. Ultrasound propagates through concrete without displaying scattering effects at frequencies less than 50 kHz and displaying scattering at frequencies exceeding 100 kHz [55]. Transmitted waves recognize complex heterogeneous media as a solid media and are propagated when the generated waves are dominated by low-frequency components corresponding to 50 kHz, such as the surface waves introduced in Section 2.2. However, excitation of waves with high-frequency components over 100 kHz leads to reflection, refraction and mode conversion of waves due to a heterogeneous internal composition among cement pastes, fine aggregate, and coarse aggregates in concrete. The diffusion of an ultrasonic wave in concrete with coarse and fine aggregates and the resulting attenuation of the ultrasound energy are illustrated in Figure 4. The typical one-dimensional diffusion with dissipation and initial energy deposition terms are shown in Equation (1) [55]. The two-dimensional diffusion phenomena of ultrasounds in concrete were also studied using surface probing transducers. Aggelis and Philippidis [56] investigated the dispersion and attenuation of ultrasonic waves in mortar, subsequently discovering the effects of frequency, water-to-cement ratio and fine aggregate on dispersion characteristics.

(1) $$D\frac{\partial^{2}\langle E\left(z,t\right)\rangle }{\partial z^{2}}-\frac{\partial \langle E\left(z,t\right)\rangle }{\partial t}-\sigma \langle E\left(z,t\right)\rangle =E_{0}\delta \left(z\right)\delta \left(t\right)$$

where D and σ are the diffusion and dissipation coefficients with regard to frequency, $E_{0}$ is the initial energy, and 〈E(z,t)〉 is the measured spectral energy density at a specific time t and point z.

In et al. [27] monitored and evaluated the self-healing process in concrete using diffuse ultrasonic parameters from two-dimensional diffusion models. Tensile and flexural cracked specimens were controlled with a tolerance of less than 200 µm. Additionally, 457 mm by 127 mm by 127 mm beam specimens from an unbonded post-tensioned bar were prepared. The damaged specimens were immersed in NaCl solutions for a period of 120 days to describe self-healing processes in marine structures. Two diffuse ultrasound parameters, namely arrival time of maximum energy (ATME) and diffusivity, were measured to compare changes in crack width at the surface of concrete using microscopy measurements. The self-healing process after crack generation decreased ATME and increased diffusivity. The study results indicated that diffusivity is the most sensitive among diffuse ultrasound parameters with respect to the prediction of the self-healing process. The relationship between exposure time and diffusivity was derived using an exponential function. This exponential relationship included measured diffusivity, asymptotic (maximum) diffusivity, initial damage, and rate of self-healing as parameters.

### 2.4. Coda Wave Interferometry (CWI)

**Figure 5:**
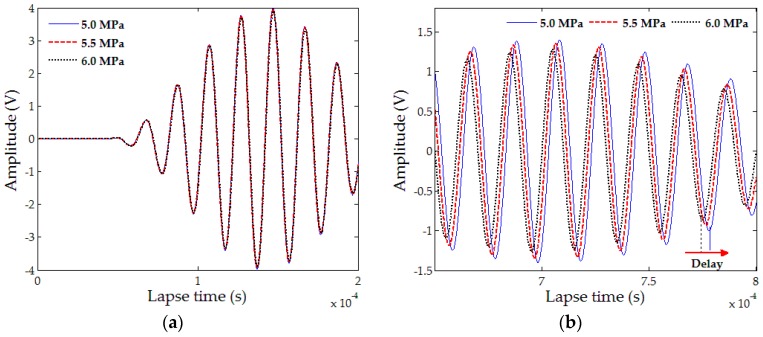
Typical ultrasonic wave signals in concrete: (**a**) earlier parts and (**b**) later parts [58].

A coda wave indicates reverberation components of randomly scattered waves due to scattering effects in heterogeneous media (e.g., concrete). The length of the scattered wave travel path exceeds that of the direct wave path and thus, the arrival time of scattering wave components (coda wave) is after that of direct wave components (ballistic wave) [57]. As shown in Figure 5, no differences exist between the three measured signals in the earlier parts. However, a small delay in the arrival signal is measured in the later parts due to changes in stress conditions from acoustoelastic effects. At this point, relative velocity change is calculated through delay time. Another parameter α is defined as a stretching parameter and studied to analyze the coda wave signal. The reference signal condition and range of analyzed time are determined to derive the stretching parameter α since its value maximizes the correlation coefficients of $CC\left(\alpha_{i}\right)$ in Equation (2).

(2) $$CC\left(\alpha_{i}\right)=\;\frac{\int_{t_{1}}^{t_{2}}u_{0}\left[t\left(1-\alpha_{i}\right)\right]u_{p}\left[t\right]dt}{\sqrt{\int_{t_{1}}^{t_{2}}{u_{0}}^{2}\left[t\left(1-\alpha_{i}\right)\right]dt\int_{t_{1}}^{t_{2}}{u_{p}}^{2}\left[t\right]dt}}.$$

Here, $t_{1}$ and $t_{2}$ are the time ranges in the coda wave components and $u_{p}$ is the arrival signal. The scattering characteristics of ultrasonic guided waves change with respect to internal media conditions. Therefore, the effects of the internal media conditions can be investigated in more detail by analyzing the coda wave signal. CWI is based on differences in signals between randomly scattered wave components.

Liu et al. [28] conducted experiments to evaluate the self-healing of internal microcracks in bacteria cementitious mortars using CWI and the recovery of compressive strength. Both bacteria-added specimens and pure specimens were cured under the water and air exposure conditions over 50 days. The determination of an appropriate window size and shifted signal influenced the results related to velocity change in CWI techniques. The study results indicated that the signal was measured after 50 µs traveling internal space. The presence of bacteria agents did not affect the relative velocity in the uncracked specimens, but the relative velocity changed to 4% in the neat-sprayed cracked specimens and to 7% in the bacterial-sprayed cracked specimens due to self-healing. Hilloulin et al. [29] applied a nonlinear ultrasound coda wave to monitor autogenous healing in cementitious materials with image-based analysis techniques, based on their previous test results that CWI discriminated different crack volumes with a very good sensitivity [59]. In the study, the stretching parameters were used to monitor the self-healing state, with the changes in stretching parameters indicating the differences in self-healing performance between mix proportions. Additionally, a stretching parameter was derived as the most sensitive parameter that could monitor the self-healing process among parameters from diffusion ultrasound phenomena in concrete.

### 2.5. Acoustic Emission (AE)

**Figure 6:**
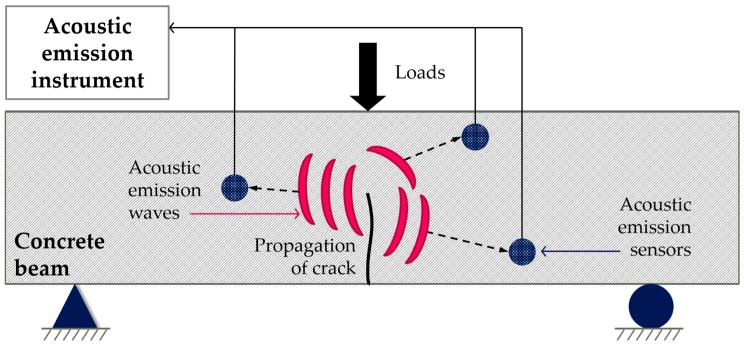
Measurements of acoustic emission (AE) signals for crack propagation.

The deformation or failure of solid media leads to the detection of generated sound (elastic wave) through AE sensors (Figure 6). The generated sound is then evaluated through the point of nondestructive tests and defined by AE testing. Specifically, AE testing can detect and predict failure of materials and structures since it monitors and inspects the propagation of microcracks and small deformation in materials prior to failure. Two types of AE signals are detected in AE sensors. First, a burst AE signal is detected due to yielding, deformation, dissolution, solidification, cracking, and fracture failure of materials. Second, a continuous AE signal is detected due to friction and leakage on the crack surface. Generally, AE technique is used to detect leakage of gas in pipe and shell structures in the fields. Additionally, the evaluation of corrosion in reinforced concrete structures is studied.

In the previous studies, Granger et al. [39,40] monitored the autogenous healing of ultra-high performance concrete through time-reversal techniques. The authors confirmed that there was decreased energy and amplitude during the cracking process in addition to regained energy and amplitude during the healing process. Additionally, AE is generally used to evaluate autonomous crack healing using encapsulated healing agents [41,42,43,44,45,46]. AE tests are conducted with flexural tests and combined with digital image correlation to identify crack generation and breakage of embedded capsules. Van Tittelboom et al. [41] classified emission energy from acoustic sounds into different types ranging from Class 1 to Class 7 and plotted the emission energy with respect to the load-displacement curve. Tsangouri et al. [42] investigated autonomous crack healing mechanisms by encapsulated healing agents in concrete under flexural damage. Crack generation was visualized through digital image correlation (DIC) while the breakage of embedded macro-capsule was evaluated by acoustic emission (AE) analysis. In this study, the damaged matrix and rupture of capsules was classified through AE analysis. Karaiskos et al. [44] monitored the performance of autonomously healed large-scale concrete beams in which the support span was 2800 mm. Van Tittelboom et al. [45] evaluated the survivability of capsules during mixing and its breakability during crack generation with digital image correlation and X-ray radiography to evaluate the efficiency of new encapsulation methods. The rupture of capsules to release healing agents at the initiation of self-healing was detected with the point of rupture estimated by using three-dimensional localization based on the arrival times at different sensors [46].

## 3. Applicability and Limitation of Ultrasonic Methods

In previous studies, various self-healing assessment methods have been classified based on objectives and characteristics [7,8,9]. Infrared thermography, radiography and measurements of electrical resistance are also used to detect cracks, voids, delaminations and damage in concrete. However, this section focuses on ultrasonic nondestructive tests using various assessment methods due to both absence of existing research examining the evaluation of self-healing concrete using other techniques.

In previous reviews, Van Tittelboom and De Belie [7] analyzed various assessment methods to evaluate the regained mechanical and durability properties of self-healing concrete. Assessment techniques were classified based on the following characteristics: visualization and determination, tightness and mechanical properties. Tang et al. [8] summarized various evaluation methods to evaluate self-healing efficiency in cementitious materials and suggested four independent criteria, namely reliability, quality of results, operational consideration and in-situ applicability. Muhammad et al. [9] classified the test methods as macro-scale (e.g., loading tests), micro-scale (e.g., scanning electron microscope, X-ray powder diffraction) and nano-scale (e.g., evaluations of interfacial transition zones) tests.

In this study, for the discussion of applicability and limitations, in-situ applicability is adopted as a criterion and four additional criteria are defined as follows: evaluation of the change in crack size, evaluation of regained durability properties, evaluation of regained mechanical properties and appropriate self-healing agents. First, self-healing was evaluated and verified through the most intuitive criteria, namely microscopic observation to monitor changes in crack width at the surface. Furthermore, the maximum self-healing performance of each self-healing agent was defined as the ratio of the initial crack width at the surface to that of the fully closed crack. However, it was not possible to measure a crack width in a specific location since crack width is not constant along a crack. Therefore, the change in crack size could also be considered as crack depth. Second, the ultimate goal to develop self-healing technology for concrete structures is to improve the durability properties. It is not possible to apply current lab-scale durability evaluation methods, such as permeability and chloride-ion diffusivity tests, on concrete structures in operation. Therefore, it is necessary to suggest appropriate nondestructive techniques to evaluate durability properties in various fields. Third, existing research focused on regained mechanical properties, although the efficiency of recovery of mechanical properties is considerably lower than the performance of regained durability properties and crack sizes. Fourth, various self-healing materials possess different mechanisms to seal cracks. Therefore, it is necessary to consider different self-healing mechanisms, effectiveness of fit between techniques, and self-healing agents. Fifth, the ultimate goal of studying nondestructive tests as assessment techniques for self-healing concrete is to provide effective evaluations and maintenances without any interruption in the structures. Finally, the limitation of each nondestructive technique for evaluating self-healing concrete is discussed. In this classification, criteria are evaluated with respect to four grades, studied in previous literature, must be able to apply, might be able to apply and might not be able to apply.

### 3.1. Evaluation of Change in Crack Size

One of the most widely studied parameters for the evaluation of self-healing performance is crack width, which is typically measured via microscopic observations. In recent years, image scanning-based techniques have emerged as a potentially efficient approach for the measurement of surface crack width. The estimated results of crack width by image scanning are quite similar to those by microscopic measurements. Therefore, it may not be meaningful to develop another method for crack width estimation using ultrasonic techniques. The present review focuses on the other crack size indices, including crack depth, with the summarized results shown in Table 1.

The change in the global volume of cracks could be one of the most suitable indices for the evaluation of self-healing performance. However, there are no reliable ultrasonic test methods capable of estimating the global crack volume to date. Therefore, discussions for the change in global crack volume due to self-healing are excluded from the scope of this paper.

**Figure 7:**
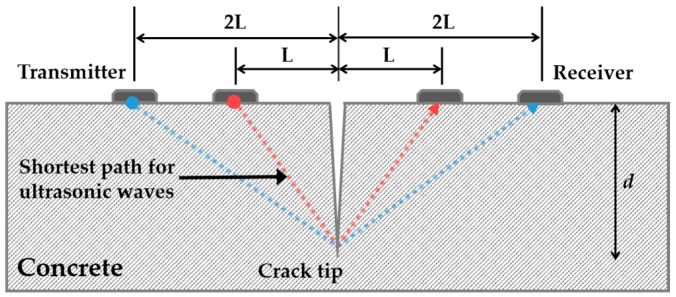
Concepts of time-of-flight diffraction (TOFD) methods.

First, the review focuses on the recovery of crack depth [14,15,30] using TOFD methods. In these methods, crack depth is determined using the velocity of longitudinal waves that corresponds to the fastest arrival signal, length between two transducers and transmission times. Figure 7 illustrates the basic principle of crack depth estimation using ultrasonic transducers attached through indirect methods. Crack depth d can be estimated through Equation (3) using the distance L between transducer and crack. Furthermore, TOFD methods are used as nondestructive techniques to characterize cracks in concrete structures prior to the application on self-healing concrete. However, the application of TOFD methods on concrete structures to identify crack depth has several disadvantages in addition to an unacceptable error range due to crack tips that are not clearly defined in concrete. Additionally, the crack-filling process resulting from self-healing is assumed to be as follows. Crack is filled from the crack tip to crack surface or the crack surface is closed first and the crack-filling material is filled from the crack tip. Neither assumption considers the case of partially closed cracks (Figure 2). The fastest arrival signals are passed through crack-filling materials when a crack is closed in the middle of the specimen. It is then impossible to monitor the self-healing process occurring in the crack tip with respect to the first filled materials at the middle since the change in transmission time is not shown.

(3) $$d=\sqrt{\frac{4{t_{L}}^{2}-{t_{2L}}^{2}}{{t_{2L}}^{2}-{t_{L}}^{2}}}$$

where $t_{i}$ indicates the measured time when the distance between transducer and crack is i.

Second, it is important to study surface-wave application to estimate the recovery of crack depth, despite the lack of existing research on this topic. This is the result of crack-depth estimation methods using surface-wave transmission having considerably more sensitive characteristics when compared to pulse velocity. As mentioned in Section 2.2, waves are propagated along a surface within the length of wavelength from the surface. Therefore, the transmission of surface waves depends on crack depth. To estimate crack depth using the transmission of surface waves, Angel and Achenbach [60] proposed theoretical solutions for the transmission and reflection of surface waves across a single crack with regard to the normalized crack depth (crack depth divided by wavelength). Transmission coefficients are applied to determine the crack depth in concrete [61,62,63,64]. Significant differences are not observed between transmission coefficients in artificial single cracks generated by notches and real cracks generated by three-point bending tests [61]. Additionally, crack width does not affect the transmission of surface waves [61]. Kee and Zhu [62] suggested that a normalized crack depth that is smaller than 0.3 corresponds to a sensitive useful range for crack depth estimation based on normalized transmission coefficients for different excitation frequencies. Meanwhile, Shin et al. [63] suggested a spectral energy-based approach to estimate crack depths in concrete in order to neglect dependent characteristics of transmission coefficients to frequency. In addition, the transmission of surface waves across multiple distributed surface-breaking cracks was examined [64]. Additionally, when a crack is partially closed from the external compressive forces, the self-healing process can be successfully monitored using transmission coefficients since transmission coefficients are sensitively changed in the process of crack closing when compared with the group velocity [65]. Therefore, the transmission of surface waves can be successfully applied in both self-healing mechanisms, namely self-healing from the crack tip and irregular self-healing at the middle of the crack depth.

Third, the crack depth may be estimated by locating the crack tip through the localization of AE in which the distance to the crack tip from each sensor is determined using different arrival times at multiple sensors as mentioned in Section 2.5. Therefore, AE analysis could correspond to one of the most effective nondestructive techniques to confirm the change in crack depth due to self-healing, as suggested by previous fracture mechanics studies [44]. However, AE analysis may need to be always accompanied by destructive loadings and a consequent propagation of cracks. Therefore, it could be difficult to evaluate the recovery of crack depth from healing materials without fracture processes.

Fourth, the evaluation of crack depth using diffusion phenomena of ultrasound in concrete is examined, although the history of nondestructive techniques using diffuse ultrasound in concrete is not intensively studied when compared with pulse velocity and surface-wave transmission [66,67,68,69,70]. The experimental results of artificial crack depth generated by notches are similar to the numerical simulation results [66]. The diffusion of ultrasound in concrete with different types of artificial notches like non-vertical cracks and two parallel cracks were investigated [67]. The ATME is suggested as the best indicator to estimate crack depth created by notches [66,67,68]. However, an evaluation parameter appropriate for real cracks is not suggested [68]. The effects of crack morphology on the diffusion of ultrasound are discussed using numerical simulations [69]. Simultaneously, closed cracks are simulated through the shaker, with the correlation between crack depth and changes in the ATME then being examined [70]. The crack morphology significantly affects the diffusion of ultrasound in concrete [68,69,70]. The performance evaluation on crack tip is successfully applied using diffuse ultrasound when self-healing processes are sequentially initiated from the crack tip. However, the efficiency of the diffuse ultrasound technique is considerably reduced due to conditions that differ from the morphology of the internal crack surface when self-healing materials fill the crack at the middle.

Finally, crack depth estimation using CWI is not intensively studied as compared to that using pulse velocity, surface wave, and diffuse ultrasound. Therefore, the possibility of applying CWI techniques to self-healing concrete should be carefully considered. The measured coda wave signal scattered in concrete internal structures will change due to the self-healing process [28,29]. Additionally, crack-filling materials shorten the lag time [29]. Simultaneously, crack detection in cementitious materials using coda wave components was examined [59]. In this study, the estimated crack volume exhibited a linear relationship with the coefficient α and the change in the relative velocity in case of crack width within one hundred of microns [59]. The sensitive characteristics of CWI parameters to the change in crack volume within small widths may emerge as a promising approach to evaluate the performance of self-healing, since self-healing technologies are limited to small crack widths. However, neither crack width nor depth has so far been suggested as an evaluation parameter sensitive to the change of coda wave components. Therefore, further study would be valuable to evaluate the crack depth using coda waves. It can be replaced via a comprehensive evaluation using crack volume that combines the concepts of crack width and depth on self-healing concrete.

### 3.2. Evaluation of Regained Durability Properties

The nondestructive evaluation of the durability properties of in-situ concrete structures was rarely required until recent years, and thus few previous studies tried to investigate relationships between the durability properties and NDT parameters. However, it is necessary to perform research to evaluate the increase in durability regained in the process of self-healing with the development of self-healing technologies. Most previous studies have evaluated regained durability properties including water permeability [12,16,25,26,32], air permeability [47] and ion diffusivity tests [23,24,32]. Generally, nondestructive test methods support the results of durability tests for self-healing concrete in different specimens with the same mix proportions and healing agents [16,23]. However, evaluations of the regained durability properties and changes in nondestructive test parameters on the same self-healing concrete specimen were not examined frequently [24,26].

In addition, research on the estimation of durability properties via ultrasonic nondestructive test methods has only examined the correlation between gas permeability and pulse velocity. First, the correlation model between ultrasonic parameters and gas permeability of cementitious materials was investigated [71]. In their study, different water contents were used and the water-to-cement ratio varied. Pulse velocity and ultrasonic attenuation were investigated as ultrasonic parameters. A linear regression curve with higher regression coefficients between pulse velocity and gas permeability was derived.

The regain in the durability properties due to self-healing may be evaluated by examining the microstructural characteristics of concrete. Various ultrasonic techniques have been attempted to characterize the microstructure of concrete [72,73,74,75]. These studies examined the relationship between Rayleigh wave velocities and capillary porosities in cementitious materials with different water contents [73]. Therefore, change in the linkages between the pores due to self-healing in concrete might be able to be monitored through measurements of R-wave. Diffuse ultrasound and CWI are highly promising techniques to predict durability properties of concrete because these techniques are based on the scattering effects in concrete. The dissipation phenomena of diffuse ultrasound due to material attenuation are dominated by a cement paste matrix as opposed to ITZ (interfacial transition zone) [74]. Furthermore, the amount of energy dissipation exhibits a linear relationship with frequency [75]. The prediction of air content through diffusivity was examined and revealed a good fit. Microcrack damages from alkali–silica reactions and thermal damages were evaluated using a diffusivity index [76]. Additionally, the prediction of setting time in concrete using diffuse ultrasounds was examined [77]. Sensitivity characteristics between changes of material properties of concrete and the diffusion of ultrasound were verified through previous studies.

However, as indicated in Table 2, it is difficult to apply AE analysis to monitor the changes in water and air permeability and chloride-ion diffusivity because AE phenomena only occur when a structure undergoes a fracture process. Additionally, there is an absence of previous studies investigating the effects of the change in microstructure of cementitious materials on AE parameters.

### 3.3. Evaluation of Changes in Mechanical Properties

A few studies have focused on the regained mechanical properties in the process of self-healing to evaluate the performance of developing agents [11,13,17,18,21,22,25,28,30,33,34,39,40,41,42,43,44,45,46]. Among these, damage and healing indices have been defined as mechanical property indices and their correlations have been analyzed [11,17,25,33,34]. Additionally, damage index and healing ratio have been defined using the NDT parameter. The correlations between degree of damage and healing ratios using compressive strength and UPV have been analyzed [33]. Meanwhile, several researchers have studied the correlation between flexural strength and AE parameters [41], as indicated in Table 3.

In general, concrete has unique characteristics that show a strong correlation between strength and stiffness. Thus, many concrete design codes worldwide (e.g., Eurocode, ACI 318) specify the relationship between the strength and stiffness of concrete. Accordingly, regain in concrete strength in previous NDT studies was predicted using the estimation of the regain in stiffness. However, the development of strength recovery due to self-healing was frequently found to be relatively small, except for certain capsule-based self-healing [38,39]. Therefore, in this study, the evaluation of the regain in mechanical properties focuses on the evaluation of regained stiffness.

Regained mechanical properties were evaluated using AE analysis with respect to flexural strength, stiffness and AE parameters in self-healing concrete [39,40,41,42,43,44,45,46]. The results indicated that the number of events and intensity of energy increased in the reloading states when the recovery of flexural strength and stiffness increased. The following assumptions could be the prerequisites to monitor the regained mechanical properties through nondestructive tests. When a crack is propagated through retests after self-healing, it is necessary for the location of crack propagation to be generated in the same direction from the initial tests. At this time, the coefficients of fracture tests and AE sounds can be compared with the effects of self-healing.

The relationship between mechanical properties and pulse velocity was investigated. First, UPV is the most widely used nondestructive technique to evaluate mechanical properties through standard code. Generally, compressive strength is directly related to stiffness, which is related to tightness. These relationships aided in deriving a strength prediction model using pulse velocity based on the estimation of stiffness and tightness. An increase in the compressive strength at an early age in the process of curing was monitored through R-wave velocities [78,79]. The elastic properties of concrete were evaluated using surface waves with the results indicating a high degree of agreement with those of previous empirical solutions [80]. Therefore, the wave velocity-based methods might be appropriate to evaluation of regained mechanical properties.

In contrast, the application of regained mechanical properties in the process of self-healing as well as the monitoring of the development of concrete strength using diffuse ultrasound phenomena are not examined to date. A study could first monitor the development of compressive strength and stiffness of concrete at an early age. This could be followed by applying diffuse ultrasound and CWI to evaluate the regained mechanical properties in the process of self-healing.

### 3.4. Self-Healing Assessment for In Situ Structures

The evaluation indices, occurrence of crack damage, effects of environmental conditions and standard criteria should be reviewed as shown in Table 4 to apply assessment techniques for in-situ structures. The absence of standardized test methods can cause errors from different operational conditions and the distortion of test results. Standard test methods are not established, except for UPV measurements. Other assessment techniques rely on the software built in the equipment or analyzed through guidelines introduced in previous studies. This indicates that there are potential improvements with respect to the analysis progress.

Several types of evaluation indices are used in each technique. In the case of UPV and CWI, an evaluation index is dependent on the other evaluation indices (e.g., transmission time and P-wave velocities). Therefore, only one measured index is used for evaluation of maintenance and inspection of damage in concrete structures. In contrast, specific evaluation indices are independent on the other parameters in surface-wave transmission, AE, and diffuse ultrasound. Therefore, in the case of surface-wave transmission, AE and diffuse ultrasound, the most sensitive index is determined first to establish standard test methods toward future practical application on the real structures.

The effect of various environmental conditions also constitutes an important issue in the application of in-situ structures. Therefore, the authors classify the environmental effects into three states: major, moderate and minor. The measurements of pulse velocity are dependent on the moisture conditions and water content. The change in R-wave velocities is very low when compared to P-wave velocities due to the change in moisture conditions. The current study concludes that the transmission of surface wave corresponds to minor effects with respect to the environmental conditions. Conversely, an analysis of the AE signals is dependent on environmental noises. Therefore, the determination of threshold to neglect the environmental noise signals is an important factor. For example, in a lab-scale evaluation, the sounds from the universal test machines are also measured in the software and should be recognized as noises. The diffusion of ultrasound in concrete is also affected by the changes in environmental conditions, such as the influence of temperature and coupling condition between transducers to surface. However, the relative velocity change in ordinary temperature is lower than 1%. Effects from the moisture conditions and water contents in concrete are not clearly investigated to date.

In summary, UPV and AE sounds were applied on in-situ structures to detect damage and they have been tried to evaluate self-healing performance in large-scale concrete beam [44]. The possibility of application in in-situ structures is summarized in Table 5.

### 3.5. Applicability for Different Self-Healing Agents

The measurements of pulse velocity, transmission coefficients, diffusion parameters and characteristics of coda wave can be applied in all types of self-healing agents. The nondestructive evaluated performance through each self-healing agent is listed in Table 6. In this section, applications of various self-healing agents are discussed from the viewpoint of self-healing agents and nondestructive evaluation methods.

First, UPV is applied to evaluate self-healing performance using autogenic self-healing, chemical agents, bacteria, microcapsules, and macrocapsules [11,14,15,16,17,18,22,30,48]. In the aforementioned previous studies, changes in transmission time or the other pulse velocity indices are reported due to all types of self-healing agents. Therefore, it can be inferred that pulse velocity index experiences a slight change when self-healing materials fill the internal crack surface.

Second, transmission coefficients are used to evaluate an autogenic self-healing process [26]. Although transmission coefficients were only used for the self-healing form continued hydration, transmission coefficients can be applied to other types of self-healing agents. This is due to propagated waves having the ability to recognize self-healing agents, such as chemical agents, bacteria and microcapsules, as being part of concrete structures. Small scattering effects from the presence of self-healing agents correspond to minor effects when compared with heterogeneous characteristics of concrete due to coarse aggregates.

Diffuse ultrasound and CWI techniques are affected by the internal microstructure of concrete. Therefore, measured signals can change from the process of crack filling using various self-healing materials in identical concrete compositions. From this viewpoint, the development of comprehensive evaluations on the performance of self-healing between various agents is desired. Although diffusion and dissipation indices can experience slight changes due to the types and contents of self-healing agents, it may be concluded that the effects of substituting self-healing agents such as bacteria are negligible for some parts of cement pastes and aggregates, when compared with the effects of changes in mix proportions.

Finally, AE analysis is not appropriate for general self-healing mechanisms due to the occurrence of low slight acoustic signals from the crack-filling materials when compared to crack propagation. AE-based evaluation for self-healing concrete is suitable for comparison studies in the process of improving the performance of capsule-based self-healing element technology or the technologies that can make recovery of mechanical properties.

### 3.6. Limitations

Finally, the limitations of each nondestructive technique for the evaluation on self-healing concrete are discussed in Table 7. Measurements of pulse velocity are most frequently used as nondestructive test methods to evaluate the characteristics of crack, durability properties, mechanical properties and self-healing performance. Although UPV has an advantage with respect to convenience, there are some limitations on its use in future practical applications. First, monitoring partially closed cracks due to self-healing through UPV does not result in reliable evaluations. Second, velocity-based parameters are significantly affected by environmental conditions. Additionally, the estimated strength and stiffness are affected by tightness as well as moisture conditions. The effects of water content on the propagation velocity of ultrasonic pulse were investigated [81]. The target structure of self-healing concrete is similar to that of a tunnel and a dam in which sufficient water is supplied due to water leakage. It is not appropriate to apply the UPV method in the water-supplied conditions needed to realize the self-healing process. However, some self-healing technologies, such as tubular or vascular capsule systems, do not involve the use of water in the healing process. Therefore, the UPV method can be utilized in such cases with no trouble.

When surface-wave transmission is used as a nondestructive evaluation method, assessment is based on the characteristics of Rayleigh waves that transfer cracks with lengths lower than the wavelength and reflect cracks that are longer than the wavelength. In order to evaluate crack properties through surface-wave transmission, it is necessary for the specimen height to exceed the wavelength of impact sources to recognize the specimen as a half-infinite solid media. Therefore, specimen size is considerably large for lab-scale evaluations when the side and bottom reflections of propagated waves are considered. Additionally, improved surface-wave transmission measurement techniques applying non-contact air-coupled sensors were studied to avoid a coupling problem between the concrete surface and contact accelerometers [64,65]. Currently, a free drop of a steel ball with various diameters to change contact time and wavelength of waves is used to generate surface waves. Excitation methods using transducers with power amplifiers were studied since previous surface-wave excitation using a free drop of a steel ball involves a manualized method [82]. In the experiments using transducers, it must be expected to retain the advantages of higher signal consistency in specific frequency regions.

As shown in Table 1, Table 2, Table 3, Table 4, Table 5 and Table 6, the application fields of AE analysis are quite limited. The occurrence of an AE event can detect the breakage of macrocapsules and locations of defects. However, other damage conditions (e.g., degradation of durability properties) could not be estimated through AE analysis with the exception of propagation cracks. The quality of experimental results from data processing and analysis in acoustic AE signals is mostly dependent on the ability of proficient technicians. Additionally, AE is not adequate with respect to structures subjected to noise pollution since the AE signal is sensitive to changes in external conditions such as consistent noises. Similarly, a standard test method is not established, and the measured signal is analyzed through built-in software supported by each manufacturer. The AE analysis is appropriate in lab-scale performance evaluation of self-healing concrete using capsules to locate the breakage of capsules. Therefore, AE can only be applied to selected self-healing element technology using capsules. It might be hard to evaluate the self-healing capability of bacteria or other chemical admixtures based on repaired concrete.

Currently, excitations through transducers with wide and high frequency ranges are typically used to simulate the diffusion phenomena of ultrasound in concrete based on scattering effects between matrices and aggregates. Concrete is a relatively heavy loss material from the viewpoint of attenuation of waves among construction building materials. Thus, it is necessary to insert an amplified signal into transducers to measure output signals with reasonable amplitude. Furthermore, there are several diffusion parameters including dissipation, diffusivity and ATME. It is necessary to determine the indices that are appropriate for evaluating different damage conditions and recovery of cracks, pores or durability. In addition, variability of measured diffusivity is large, although diffusivity exhibits the most ideal behavior to monitor internal changes and self-healing results.

CWI techniques are also based on diffusion phenomena. However, the variability of coda wave parameters is considerably small when compared to diffusivity. Relative velocity change and stretching parameters are used as indices of CWI techniques. The analyzed time ranges differ across studies. The differences from the analyzed time domain do not significantly affect the results of stretching parameters. However, the standardization of the analyzed region is necessary to improve the reliability of the test results.

## 4. Future Directions

The results of analysis of the applicability and limitation of nondestructive assessment techniques have suggested that the development of correlation model between self-healing indices (e.g., crack size, permeability) and nondestructive test parameters (e.g., pulse velocity, diffusivity) should be initially examined, followed by performing a verification of the mock-up structures.

First, the evaluation of change in crack size focusing on crack depth is discussed in Section 3.1. It is necessary for the crack recovery model to consider the change in crack depth. The present study recommends the following steps to estimate a crack-depth recovery model. Surface-wave transmission is first performed, followed by diffuse ultrasound. Finally, CWI is performed. The most sensitive behavior across a crack can be monitored through the transmission of surface waves. Additionally, the propagation of surface waves is less influenced by other factors, such as self-healing agents, environmental conditions and mix proportions. The morphology of a crack surface directly affects the diffusion of scattered waves. Therefore, supplementary evaluation methods including image-based three-dimensional CT techniques are necessary to confirm the effects of morphology of crack surface on diffuse ultrasound parameters. Previous studies on correlation between crack volume with smaller crack width and coda wave parameters are also involved. In contrast, UPV is the most general, easy to operate, and difficult to expect develop some special things in technical improvement points.

Second, existing research did not reflect the ultimate goal of developing self-healing concrete. Previous studies that focused on self-healing element technologies involved a limited and concentrated target performance of crack filling. It is not possible to expect that all material properties were regained to levels prior to the occurrence of damage. Therefore, self-healing efficiency defined by regained durability properties should be studied. In the previous studies, the water permeability in concrete was significantly influenced by the initial crack width, roughness of crack, and microstructure (e.g., pore condition). The roughness of crack surface may affect the self-healing process to form crack-filling materials between water and cement paste [83]. In addition, the chloride-ion diffusivity in concrete is likely affected by conditions of the internal crack surface as well as the microstructure. However, previous research did not investigate the effects of pore structures in concrete on self-healing performance. The present study recommends the following steps to evaluate a durability recovery. First, the diffuse ultrasound is performed, before being followed by CWI. Although both these techniques are sensitive to internal microstructural changes due to the scattering effects of propagated waves, the diffuse ultrasound is recommended first due to the lack of evidence for CWI from previous studies.

Third, nondestructive techniques using UPV and AE are the preferred technologies to evaluate mechanical properties. The present study recommends that AE for flexural loads is first determined before determining pulse velocity to evaluate the stiffness recovery. In contrast, there remains uncertainty as to whether the other techniques (e.g., surface wave transmission, diffuse ultrasound, CWI) might be used to evaluate mechanical properties. This is the result of a lack of existing research examining the same in conjunction with a direct correlation between strength (or stiffness) development and the nondestructive characteristics of each technique.

## 5. Conclusions

In this study, theories and case studies of five ultrasonic-based nondestructive test methods (i.e., measurement of pulse velocity, surface-wave transmission, diffuse ultrasound, AE analysis, and CWI techniques) were thoroughly examined with respect to their applicability and limitations in assessing the effectiveness and performance of the self-healing technologies developed for cementitious materials. The findings and conclusions of this study can be summarized as follows:

(1) The measurement of UPV and its transmission time is one of the most developed ultrasonic test methods and is widely used to evaluate the performance of the self-healing technologies. However, the partial closing of cracks and moisture conditions in concrete structures can affect the evaluation results of the self-healing performance by the UPV method. In addition, the velocity-based approach is sensitive to moisture conditions.
(2) With respect to the other nondestructive test methods (e.g., surface-wave transmission, diffuse ultrasound, AE analysis, and CWI), there are no standard test procedures to measure, process and analyze the test data. Thus, it is necessary to determine appropriate self-healing evaluation procedures for each test method by considering the target of self-healing performance evaluation (e.g., crack size, permeability).
(3) The diffuse ultrasound and CWI methods are based on the scattering of elastic waves between aggregates and matrices. Therefore, these techniques are suitable to assess the self-healing of internal damages in concrete that are associated with durability properties.
(4) Nondestructive evaluations of mechanical properties (e.g., strength, stiffness) are studied through measurements of either P-wave or R-wave velocity. Some researchers observed stiffness recovery in the process of self-healing. However, the range of regained strength is quite small, which raises the question as to whether it can be used as a measure for the performance of self-healing technologies. Therefore, when evaluation methods for the regain of mechanical properties are studied, it is proposed to focus on the stiffness using UPV or AE.
(5) All ultrasonic test methods with the exception of AE analysis can be applied for all types of self-healing materials ranging from chemical agents to capsule-based mechanisms. In contrast, the AE analysis can only be applied to regain mechanical properties from capsule-based self-healing materials because the technique is based on sensing the sounds of capsule breakages. Capsules are broken in the initial fracture test and the leakage of healing agents is assumed in the detected locations.

## Figures and Tables

**Table 1:** Evaluation of change in crack size due to self-healing.

Test Methods	UPV	SWT	AE	DU	CWI
Change in crack depth	● ^1^	○ ^2^	△ ^3^	○	△

^1^ ● indicates which assessment techniques are studied in previous literature; ^2^ ○ indicates which assessment techniques are not studied in previous literature and must be able to apply; ^3^ △ indicates which assessment techniques are not studied in previous literature and might be able to apply. UPV: ultrasonic pulse velocity; SWT: surface-wave transmission; AE: acoustic emission; DU: diffuse ultrasound; CWI: coda wave interferometry.

**Table 2:** Evaluation of regain in durability properties due to self-healing.

Test Methods	UPV ^5^	SWT	AE	DU	CWI
Permeability	●	○	× ^4^	○	○
Chloride ion diffusivity	○	○	×	○	○

^4^ × indicates which assessment techniques are not studied in previous literature and might not be able to apply; ^5^ ● gas permeability instead of water permeability are studied in previous literature.

**Table 3:** Evaluation of regain in mechanical properties due to self-healing.

Test Methods	UPV ^6^	SWT ^6^	AE ^7^	DU ^8^	CWI ^8^
Strength	△	△	●	△	△
Stiffness	○	○	●	△	△

^6^ velocity-based estimations are studied in previous literature; ^7^ ● flexural strength and stiffness instead of compression recovery are studied in previous literature; ^8^ △ basic research has not yet been performed to estimate mechanical properties of concrete.

**Table 4:** Considerations for application of ultrasonic methods on in-situ structures for self-healing evaluation.

Test Methods	Evaluation Indices	Need of Destructive Loading	Effects of Environmental Conditions	Standard Criteria
UPV	Transmission time P-wave velocities	×	Major	ASTM C597
SWT	R-wave velocities Amplitude Transmission coefficients	×	Minor	None
AE	AE energy Counts of released energy	○	Major	
DU	Diffusivity ATME Maximum energy Dissipation	×	Major	
CWI	Relative velocity change Stretching parameters	×	Moderate	

**Table 5:** Application on in-situ structures for self-healing evaluation.

Test Methods	UPV	SWT	AE	DU	CWI
In-situ structures	●	○	●	○	○

**Table 6:** Appropriate self-healing agents.

Recovery	Mechanisms	UPV	SWT	AE	DU	CWI
Natural	Continued hydration	●	●	●	●	●
Engineered	Chemical agents	●	○	△	○	○
	Bacteria	●	○	△	○	●
	Capsules	●	○	●	○	○

**Table 7:** Limitations of ultrasonic wave methods for self-healing evaluation.

Test Methods	UPV	SWT	AE	DU	CWI
Technical points	Dependent on environmental effects, Partially closed crack	Minimum size of specimen	Threshold, Fracture process	Variability of measured data	Determination of analyzed data
Unknown country	-	-	-	Evaluation of mechanical properties	

## References

[1] Van Breugel K. Is there a market for self-healing cement-based materials. Proceedings of the First International Conference on Self-Healing Materials.

[2] De Rooij M.R., van Tittelboom K., de Belie N., Schlangen E. (2011). Self-Healing Phenomena in Cement-Based Materials: Draft of State-of-the-Art Report of RILEM Technical Committee.

[3] Wu M., Johannesson B., Geiker M. (2012). A review: Self-healing in cementitious materials and engineered cementitious composite as a self-healing material. Constr. Build. Mater..

[4] Mihashi H., Nishiwaki T. (2012). Development of engineered self-healing and self-repairing concrete-state-of- the-art report. J. Adv. Concr. Technol..

[5] Snoeck D., de Belie N. (2015). From straw in bricks to modern use of microfibers in cementitious composites for improved autogenous healing—A review. Constr. Build. Mater..

[6] Yıldırım G., Keskin Ö.K., Keskin S.B., Şahmaran M., Lachemi M. (2015). A review of intrinsic self-healing capability of engineered cementitious composites: Recovery of transport and mechanical properties. Constr. Build. Mater..

[7] Van Tittelboom K., de Belie N. (2013). Self-healing in cementitious materials—A review. Materials.

[8] Tang W., Kardani O., Cui H. (2015). Robust evaluation of self-healing efficiency in cementitious materials—A review. Constr. Build. Mater..

[9] Muhammad N.Z., Shafaghat A., Keyvanfar A., Majid M.Z.A., Ghoshal S.K., Yasouj S.E.M., Ganiyu A.A., Kouchaksaraei M.S., Kamyab H., Taheri M.M. (2016). Tests and methods of evaluating the self-healing efficiency of concrete: A review. Constr. Build. Mater..

[10] Ahn T.H., Kishi T. (2010). Crack self-healing behavior of cementitious composites incorporating various mineral admixtures. J. Adv. Concr. Technol..

[11] Ferrara L., Krelani V., Carsana M. (2014). A “fracture testing” based approach to assess crack healing of concrete with and without crystalline admixtures. Constr. Build. Mater..

[12] Roig-Flores M., Pirritano F., Serna P., Ferrara L. (2016). Effect of crystalline admixtures on the self-healing capability of early-age concrete studied by means of permeability and crack closing tests. Constr. Build. Mater..

[13] Wang X., Xing F., Zhang M., Han N., Qian Z. (2013). Experimental study on cementitious composites embedded with organic microcapsules. Materials.

[14] Mostavi E., Asadi S., Hassan M.M., Alansari M. (2015). Evaluation of self-healing mechanisms in concrete with double-walled sodium silicate microcapsules. J. Mater. Civ. Eng..

[15] Kanellopoulos A., Giannaros P., Al-Tabbaa A. (2016). The effect of varying volume fraction of microcapsules on fresh, mechanical and self-healing properties of mortars. Constr. Build. Mater..

[16] Van Tittelboom K., de Belie N., de Muynck W., Verstraete W. (2010). Use of bacteria to repair cracks in concrete. Cem. Concr. Res..

[17] Xu J., Yao W. (2014). Multiscale mechanical quantification of self-healing concrete incorporating non-ureolytic bacteria-based healing agent. Cem. Concr. Res..

[18] Williams S.L., Sakib N., Kirisits M.J., Ferron R.D. (2016). Flexural strength recovery induced by vegetative bacteria added to mortar. ACI Mater. J..

[19] Lee H.X.D., Wong H.S., Buenfeld N.R. (2010). Potential of superabsorbent polymer for self-sealing cracks in concrete. Adv. Appl. Ceram..

[20] Snoeck D., Dewanckele J., Cnudde V., de Belie N. (2016). X-ray computed microtomography to study autogenous healing of cementitious materials promoted by superabsorbent polymers. Cem. Concr. Compos..

[21] Herbert E.N., Li V.C. (2013). Self-healing of microcracks in engineered cementitious composites (ECC) under a natural environment. Materials.

[22] Zhu Y., Yang Y., Yao Y. (2012). Autogenous self-healing of engineered cementitious composites under freeze-thaw cycles. Constr. Build. Mater..

[23] Sahmaran M., Yildirim G., Noori R., Ozbay E., Lachemi M. (2015). Repeatability and pervasiveness of self-healing in engineered cementitious composites. ACI Mater. J..

[24] Yildirim G., Aras G.H., Banyhussan Q.S., Şahmaran M., Lachemi M. (2015). Estimating the self-healing capability of cementitious composites through non-destructive electrical-based monitoring. NDT E Int..

[25] Nishiwaki T., Kwon S., Homma D., Yamada M., Mihashi H. (2014). Self-healing capability of fiber-reinforced cementitious composites for recovery of watertightness and mechanical properties. Materials.

[26] Aldea C.M., Song W.J., Popovics J.S., Shah S.P. (2000). Extent of healing of cracked normal strength concrete. J. Mater. Civ. Eng..

[27] In C.W., Holland R.B., Kim J.Y., Kurtis K.E., Kahn L.F., Jacobs L.J. (2013). Monitoring and evaluation of self-healing in concrete using diffuse ultrasound. NDT E Int..

[28] Liu S., Bundur Z.B., Zhu J., Ferron R.D. (2016). Evaluation of self-healing of internal cracks in biomimetic mortar using coda wave interferometry. Cem. Concr. Res..

[29] Hilloulin B., Legland J.B., Lys E., Abraham O., Loukili A., Grondin F., Durand O., Tournat V. (2016). Monitoring of autogenous crack healing in cementitious materials by the nonlinear modulation of ultrasonic coda waves, 3D microscopy and X-ray microtomography. Constr. Build. Mater..

[30] Alghamri R., Kanellopoulos A., Al-Tabbaa A. (2016). Impregnation and encapsulation of lightweight aggregates for self-healing concrete. Constr. Build. Mater..

[31] Gagné R., Argouges M. (2012). A study of the natural self-healing of mortars using air-flow measurements. Mater. Struct..

[32] Maes M., Snoeck D., de Belie N. (2016). Chloride penetration in cracked mortar and the influence of autogenous crack healing. Constr. Build. Mater..

[33] Zhong W., Yao W. (2008). Influence of damage degree on self-healing of concrete. Constr. Build. Mater..

[34] Abd Elmoaty A.M. (2011). Self-healing of polymer modified concrete. Alexandria Eng. J..

[35] Turner L. The autogenous healing of cement and concrete: Its relation to vibrated concrete and cracked concrete. Proceedings of the International Association for Testing Materials, London Congress.

[36] Lauer K.R., Slate F.O. (1956). Autogenous healing of cement paste. J. Am. Concr. Inst..

[37] Jacobsen S., Sellevold E.J. (1996). Self healing of high strength concrete after deterioration by freeze/thaw. Cem. Concr. Res..

[38] Hilloulin B., Hilloulin D., Grondin F., Loukili A., de Belie N. (2016). Mechanical regains due to self-healing in cementitious materials: Experimental measurements and micro-mechanical model. Cem. Concr. Res..

[39] Granger S., Loukili A., Pijaudier-Cabot G., Chanvillard G. (2007). Experimental characterization of the self-healing of cracks in an ultra high performance cementitious material: Mechanical tests and acoustic emission analysis. Cem. Concr. Res..

[40] Granger S., Cabot G.P., Loukili A., Marlot D., Lenain J.C. (2009). Monitoring of cracking and healing in an ultra high performance cementitious material using the time reversal technique. Cem. Concr. Res..

[41] Van Tittelboom K., de Belie N., Lehmann F., Grosse C.U. (2012). Acoustic emission analysis for the quantification of autonomous crack healing in concrete. Constr. Build. Mater..

[42] Tsangouri E., Aggelis D.G., van Tittelboom K., de Belie N., van Hemelrijck D. (2013). Detecting the activation of a self-healing mechanism in concrete by acoustic emission and digital image correlation. Sci. World J..

[43] Van Tittelboom K., Wang J., Araújo M., Snoeck D., Gruyaert E., Debbaut B., Derluyn B., Cnudde V., Tsangouri E., van Hemelrijck D. (2016). Comparison of different approaches for self-healing concrete in a large-scale lab test. Constr. Build. Mater..

[44] Karaiskos G., Tsangouri E., Aggelis D.G., van Tittelboom K., de Belie N., van Hemelrijck D. (2016). Performance monitoring of large-scale autonomously healed concrete beams under four-point bending through multiple non-destructive testing methods. Smart Mater. Struct..

[45] Van Tittelboom K., Tsangouri E., van Hemelrijck D., de Belie N. (2015). The efficiency of self-healing concrete using alternative manufacturing procedures and more realistic crack patterns. Cem. Concr. Compos..

[46] Tsangouri E., Karaiskos G., Deraemaeker A., van Hemelrijck D., Aggelis D. (2016). Assessment of acoustic emission localization accuracy on damaged and healed concrete. Constr. Build. Mater..

[47] Kang C., Kunieda M. (2014). Evaluation and observation of autogenous healing ability of bond cracks along rebar. Materials.

[48] Watanabe T., Fujiwara Y., Hashimoto C., Ishimaru K. (2011). Evaluation of self healing effect in fly-ash concrete by ultrasonic test method. Int. J. Mod. Phys. B.

[49] McCann D.M., Forde M.C. (2001). Review of NDT methods in the assessment of concrete and masonry structures. NDT E Int..

[50] American Concrete Institute (ACI) (2013). 228.2R-13 Report on Nondestructive Test Methods for Evaluation of Concrete in Structures.

[51] American Society for Testing and Materials International (ASTM) (2009). C597, Standard Test Method for Pulse Velocity through Concrete.

[52] Graff K.F. (1991). Wave Motion in Elastic Solids.

[53] Aggelis D.G., Shiotani T. (2007). Repair evaluation of concrete cracks using surface and through-transmission wave measurements. Cem. Concr. Compos..

[54] Aggelis D.G., Shiotani T., Polyzos D. (2009). Characterization of surface crack depth and repair evaluation using Rayleigh waves. Cem. Concr. Compos..

[55] Anugonda P., Wiehn J.S., Turner J.A. (2001). Diffusion of ultrasound in concrete. Ultrasonics.

[56] Aggelis D.G., Philippidis T.P. (2004). Ultrasonic wave dispersion and attenuation in fresh mortar. NDT E Int..

[57] Planès T., Larose E. (2013). A review of ultrasonic Coda Wave Interferometry in concrete. Cem. Concr. Res..

[58] Shin S.W. (2014). Applicability of coda wave interferometry technique for measurement of acoustoelastic effect of concrete. J. Korean Soc. Nondestruc. Test..

[59] Hilloulin B., Zhang Y., Abraham O., Loukili A., Grondin F., Durand O., Tournat V. (2014). Small crack detection in cementitious materials using nonlinear coda wave modulation. NDT E Int..

[60] Angel Y.C., Achenbach J.D. (1984). Reflection and transmission of obliquely incident Rayleigh waves by a surface-breaking crack. J. Acoust. Soc. Am..

[61] Popovics J.S., Song W.J., Ghandehari M., Subramaniam K.V., Achenbach J.D., Shah S.P. (2000). Application of surface wave transmission measurements for crack depth determination in concrete. ACI Mater. J..

[62] Kee S.H., Zhu J. (2010). Using air-coupled sensors to determine the depth of a surface-breaking crack in concrete. J. Acoust. Soc. Am..

[63] Shin S.W., Zhu J., Min J., Popovics J.S. (2008). Crack depth estimation in concrete using energy transmission of surface waves. ACI Mater. J..

[64] Kee S.H., Zhu J. (2011). Surface wave transmission measurements across distributed surface-breaking cracks using air-coupled sensors. J. Sound Vib..

[65] Kee S.H., Zhu J. (2014). Surface wave transmission across a partially closed surface-breaking crack in concrete. ACI Mater. J..

[66] Ramamoorthy S.K., Kane Y., Turner J.A. (2004). Ultrasound diffusion for crack depth determination in concrete. J. Acoust. Soc. Am..

[67] Seher M., In C.W., Kim J.Y., Kurtis K.E., Jacobs L.J. (2013). Numerical and experimental study of crack depth measurement in concrete using diffuse ultrasound. J. Nondestruct. Eval..

[68] Quiviger A., Payan C., Chaix J.F., Garnier V., Salin J. (2012). Effect of the presence and size of a real macro-crack on diffuse ultrasound in concrete. NDT E Int..

[69] Quiviger A., Girard A., Payan C., Chaix J.F., Garnier V., Salin J. (2013). Influence of the depth and morphology of real cracks on diffuse ultrasound in concrete: A simulation study. NDT E Int..

[70] Payan C., Quiviger A., Garnier V., Chaix J.F., Salin J. (2013). Applying diffuse ultrasound under dynamic loading to improve closed crack characterization in concrete. J. Acoust. Soc. Am..

[71] Lafhaj Z., Goueygou M., Djerbi A., Kaczmarek M. (2006). Correlation between porosity, permeability and ultrasonic parameters of mortar with variable water/cement ratio and water content. Cem. Concr. Res..

[72] Goueygou M., Lafhaj Z., Soltani F. (2009). Assessment of porosity of mortar using ultrasonic Rayleigh waves. NDT E Int..

[73] Soltani F., Goueygou M., Lafhaj Z., Piwakowski B. (2013). Relationship between ultrasonic Rayleigh wave propagation and capillary porosity in cement paste with variable water content. NDT E Int..

[74] Becker J., Jacobs L.J., Qu J. (2003). Characterization of cement-based materials using diffuse ultrasound. J. Eng. Mech..

[75] Punurai W., Jarzynski J., Qu J., Kurtis K.E., Jacobs L.J. (2007). Characterization of dissipation losses in cement paste with diffuse ultrasound. Mech. Res. Commun..

[76] Deroo F., Kim J.Y., Qu J., Sabra K., Jacobs L.J. (2010). Detection of damage in concrete using diffuse ultrasound. J. Acoust. Soc. Am..

[77] Yim H.J., An Y., Kim J.H. (2016). Water depercolation of setting cement paste evaluated by diffuse ultrasound. Cem. Concr. Compos..

[78] Popovics J.S., Song W., Achenbach J.D., Lee J.H., Andre R.F. (1998). One-sided stress wave velocity measurement in concrete. J. Eng. Mech..

[79] Shin S.W., Yun C.B., Popovics J.S., Kim J.H. (2007). Improved Rayleigh wave velocity measurement for nondestructive early-age concrete monitoring. Res. Nondestruct. Eval..

[80] Kim J.H., Kwak H.G. (2008). Nondestructive evaluation of elastic properties of concrete using simulation of surface waves. Comput.-Aided Civ. Infrastruct. Eng..

[81] Berriman J., Purnell P., Hutchins D.A., Neild A. (2005). Humidity and aggregate content correction factors for air-coupled ultrasonic evaluation of concrete. Ultrasonics.

[82] In C.W., Schempp F., Kim J.Y., Jacobs L.J. (2015). A fully non-contact, air-coupled ultrasonic measurement of surface breaking cracks in concrete. J. Nondestruct. Eval..

[83] Picandet V., Khelidj A., Bellegou H. (2009). Crack effects on gas and water permeability of concretes. Cem. Concr. Res..

